# Remote work frequency and loneliness among United Kingdom employees: the moderating role of active and passive social media use

**DOI:** 10.3389/fpsyg.2026.1930871

**Published:** 2026-08-24

**Authors:** Deshan Chen, Qiongwei Wang, Yaguang Yan

**Affiliations:** 1 Institute of Population Research, Peking University, Beijing, China; 2 School of Government, Beijing Normal University, Beijing, China; 3 Tohoku University, Sendai, Japan

**Keywords:** active social media use, loneliness, passive social media use, remote work frequency, United Kingdom

## Abstract

**Objective:**

This study examines the relationship between remote work frequency and loneliness and assesses the differential moderating effects of active (e.g., posting content) versus passive (e.g., scrolling or browsing content) social media use.

**Methods:**

Using data from Waves 13 and 14 (2021–2024) of the UK Household Longitudinal Study (UKHLS), comprising 7,601 individuals and 15,202 person-wave observations, this study employs fixed-effects regression models.

**Results:**

The findings reveal that higher remote work frequency is positively associated with greater loneliness (*β* = 0.059, 95% confidence interval [0.018, 0.100]). Furthermore, active social media use significantly buffers this positive association (*β* = −0.016, 95% confidence interval [−0.031, −0.001]), whereas passive social media use shows no significant moderating effect (*β* = −0.003, 95% confidence interval [−0.022, 0.015]).

**Conclusion:**

Greater remote work frequency is associated with increased loneliness, but active social media use attenuates this association while passive use does not. As remote work becomes more prevalent, active rather than passive social media engagement may help offset the loneliness associated with this work arrangement.

## 1 Introduction

Loneliness is increasingly recognized as a critical public health concern with profound implications for individuals and society. Defined as a distressing experience arising from quantitative or qualitative deficiencies in one’s social relationships (Perlman and Peplau, 1981), loneliness poses substantial risks that range from individual health consequences, such as increased cardiovascular disease and mortality (Holt-Lunstad et al., 2015; Hu et al., 2025; Vallée, 2025), to broader societal challenges, including weakened social cohesion and heightened healthcare burdens for governments (Meisters et al., 2021; Swader, 2019). Given its severity and widespread prevalence in modern society, understanding the factors that lead to loneliness and identifying protective factors have become an urgent priority for scholars and policymakers.

With the rapid global expansion of remote work, especially following the COVID-19 pandemic, work location has become a potentially important factor associated with loneliness among working adults. Recent studies have begun to examine the association between remote work and loneliness, yet empirical findings remain inconsistent. While some evidence suggests that remote work exacerbates loneliness through increased role overload (Fostervold et al., 2024; He et al., 2026), other studies report null effects (Oh et al., 2025). These mixed findings likely reflect methodological limitations, particularly the reliance on small, cross-sectional samples that constrain causal inference (Wright, 2025). Consequently, a rigorous investigation of the relationship between remote work and loneliness is needed.

While direct evidence linking remote work to loneliness remains limited, even less is known about the factors that may buffer or alleviate this relationship (Wright, 2025). Recent empirical efforts have begun to explore the boundary conditions of this link; for instance, Delanoeije and Verbruggen (2024) demonstrated that offline environmental and lifestyle factors, such as dog caregiving and access to outdoor space, can buffer the daily loneliness associated with teleworking. Nevertheless, existing research has focused primarily on physical and offline features of the home-working environment, leaving the potential role of virtual social interactions largely unexamined. This omission is theoretically important because remote work can reduce opportunities for spontaneous face-to-face workplace interactions without necessarily eliminating employees’ capacity to maintain relational closeness. The concept of perceived proximity and the associated far-but-close paradox suggest that geographic separation does not inevitably translate into psychological or relational distance (Wilson et al., 2008). Rather, geographically dispersed coworkers may still feel close when communication and shared identification enable them to sustain a subjective sense of connection. From this perspective, the social consequences of remote work may depend less on physical distance itself than on whether employees can preserve relational closeness through alternative channels of interaction. In an increasingly digitally mediated work environment, social media serves as an important means of sustaining interpersonal ties and mitigating social isolation (Cauberghe et al., 2021; Latikka et al., 2022). Accordingly, social media may provide one such channel by enabling employees to maintain interpersonal contact and social connection despite reduced opportunities for face-to-face interaction.

However, evidence regarding the effects of social media use on well-being remains mixed. Some studies suggest that social media communication can enhance remote workers’ perceived support from colleagues and sense of community (Pekkala et al., 2025), whereas others link generic social media use to emotional distress during the COVID-19 pandemic (Yue et al., 2023). These inconsistent findings may partly stem from a tendency to conceptualize social media use as a single, uniform behavior (Cho et al., 2025; Wu-Ouyang, 2026); as scholars have argued, the psychological consequences of digital technology depend on how it is used (Verduyn et al., 2017). Accordingly, the compensatory role of social media may depend on the specific mode of engagement. Recent qualitative evidence among digital nomads suggests that real-time interaction with close ties can alleviate loneliness, whereas passive browsing tends to intensify social comparison and exacerbate feelings of distress (Miguel et al., 2025). Despite this insightful finding, few quantitative studies have examined whether these distinct patterns of social media use interact with remote work frequency to shape employee loneliness.

To address the above gaps, the present study utilizes nationally representative longitudinal data from the United Kingdom to examine the effect of remote work on loneliness and the moderating role of different patterns of social media use. The United Kingdom provides a particularly relevant context. The country’s large service sector makes a substantial proportion of jobs technically suitable for remote work, while its prolonged pandemic restrictions accelerated organizational investment in remote-working practices and normalized working from home (Parry, 2025). An important feature of remote work is that it aligns with a British work culture increasingly organized around responsible autonomy, whereby employees gain greater discretion over how and where they work in exchange for reciprocal trust with managers, and managerial control shifts from monitoring physical presence toward output-based expectations (Abgeller et al., 2024). This arrangement also operates within a regulatory environment that recognizes workers’ heightened expectations of privacy at home: employers may monitor work-related internet and device activity, but such monitoring must be lawful, necessary, proportionate, and minimally intrusive. Against this institutional backdrop, remote work is unusually prevalent: full-time, university-educated employees in the United Kingdom worked remotely for an average of 1.8 days per week in 2025, the second-highest level among the countries surveyed after Canada (Aksoy et al., 2025). Moreover, the United Kingdom is characterized by a highly connected but increasingly passive social media use environment, with the proportion of adult social media users who actively posted, shared, or commented falling from 61% in 2024 to 49% in 2025 (Ofcom, 2026). Together, these institutional and cultural conditions make the United Kingdom a particularly relevant setting for examining whether different forms of social media use shape the relationship between remote work and loneliness.

This study has two primary objectives corresponding to our hypotheses. First, we aim to clarify the main effect of remote work frequency on loneliness in the United Kingdom. Second, we seek to differentiate between distinct social media use patterns to identify which specific form of usage effectively buffers the relationship between remote work and loneliness. By doing so, this research moves beyond correlational insights to offer robust evidence on how specific digital social media use patterns shape loneliness in the modern remote work context.

## 2 Literature review and hypotheses

### 2.1 Remote work and loneliness: a latent-deprivation account

Loneliness is generally conceptualized as an aversive and subjective experience of perceived disconnection that arises when individuals regard their social relationships as insufficient in quantity or quality (Perlman and Peplau, 1981). Loneliness and social isolation are conceptually distinct. Social isolation refers to the objective absence or limited extent of social contacts, interactions, and network ties; loneliness is the perception of social isolation or the subjective feeling of being lonely (Barjaková et al., 2023). Therefore, loneliness does not necessarily coincide with isolation: individuals may feel lonely despite being embedded in extensive social networks, whereas others may have relatively few social contacts without experiencing loneliness.

Existing research identifies a broad range of factors associated with loneliness, including demographic and socioeconomic characteristics, living arrangements, partnership status, physical and mental health, major life transitions, and the structure and quality of social networks (Barjaková et al., 2023; Hutten et al., 2022). Among these factors, relational conditions are especially important, given the inherently relational nature of loneliness. For working-age adults, the workplace constitutes a major setting in which regular interaction, social belonging, and collective engagement are developed and maintained. Changes in work arrangements that restrict access to these relational resources may therefore increase loneliness.

Jahoda’s latent deprivation theory provides a theoretical basis for understanding the relationship between remote work frequency and loneliness. Developed from research on mass unemployment in the 1930s, the theory distinguishes the manifest function of employment, namely income, from five latent functions that support psychological well-being: time structure, social contact, collective purpose, status and identity, and regular activity (Jahoda, 1982; Jahoda et al., 2017). From this perspective, the psychological consequences of unemployment arise not only from income loss but also from the withdrawal of these latent functions.

Although originally developed to explain unemployment, the theory can also be extended to variations in access to latent functions among people who remain employed (Batinic et al., 2010). Remote work represents a distinctive form of partial latent deprivation within employment. Remote employees retain their jobs and therefore continue to receive income, occupational identity, collective purpose, and a basic structure to the working day. However, working from home may selectively reduce access to the latent function most directly relevant to loneliness: regular workplace social contact. As remote work frequency increases, employees spend progressively less time co-located with colleagues and have fewer opportunities for spontaneous conversations, incidental encounters, shared routines, and collective workplace activities through which everyday social connection is maintained (Simon et al., 2023; Viererbl et al., 2022; Yang et al., 2022). Although online communication may partly compensate for physical separation, it may not fully reproduce the emotional and relational qualities of face-to-face interaction. For example, virtual teams may achieve communication effectiveness comparable to face-to-face teams while reporting lower satisfaction with the interaction process (Warkentin et al., 1997). Because loneliness arises when individuals perceive their social relationships as insufficient in quantity or quality relative to the connection they desire (Perlman and Peplau, 1981), this progressive erosion of regular and spontaneous workplace social contact should leave frequent remote workers feeling less connected to colleagues and less embedded in the everyday social life of the organization, and therefore lonelier. Taken together, we propose the following hypothesis:

*H1*: A higher frequency of remote work is positively associated with loneliness.

### 2.2 Active versus passive social media use as moderators

To achieve the second objective, we explore the potential moderating roles of different forms of social media use. Before the active–passive distinction was introduced, research on social media use commonly relied on aggregate indicators, such as overall time spent. However, such measures obscure substantial differences in how users engage with social media: two individuals with similar usage time may pursue very different activities and, consequently, experience different relational outcomes. To account for these differences, Burke et al. (2010) first drew an empirical distinction between directed communication and passive consumption of others’ content, a distinction later formalized as the active–passive model (Verduyn et al., 2015; Verduyn et al., 2017). In this model, active social media use (ASMU) refers to behaviors that involve initiating, contributing to, or responding to interpersonal exchanges, including posting content, commenting, sharing, and communicating directly with other users. In contrast, passive social media use (PSMU) refers to monitoring or consuming others’ content without directly engaging with them, such as browsing news feeds or viewing others’ profiles, photos, and posts. These differences suggest that active and passive social media use may play distinct moderating roles in the relationship between remote work frequency and loneliness.

The internet-enhanced self-disclosure hypothesis (IESD) provides a theoretical foundation for understanding this relationship. Self-disclosure refers to the voluntary and intentional revelation of one’s emotions, opinions, and lived experiences (Jourard and Lasakow, 1958). On social networking sites, this exchange takes a distinctly platform-mediated form, as users convey personal information through uploaded images, status updates on their profiles, and commentary on others’ content (Chen and Sharma, 2013; Kwak et al., 2014; Lin et al., 2021). The IESD framework is based on three key assumptions: (1) online communication stimulates online self-disclosure; (2) increased online self-disclosure (rather than mere exposure to instant messaging) leads to higher-quality relationships; and (3) these higher-quality relationships, in turn, enhance adolescents’ well-being (Valkenburg and Peter, 2009). Subsequent research has provided empirical support for these assumptions (Chu et al., 2023; Luo and Hancock, 2020; Matthes et al., 2021).

Drawing on the IESD framework, particularly its second assumption, we argue that ASMU and PSMU afford different opportunities for self-disclosure, leading to differences in relationship quality that ultimately determine their capacity to mitigate the positive association between remote work and loneliness. Involving interactive behaviors such as posting, commenting, and responding, ASMU directly aligns with the core concept of self-disclosure by enabling individuals to express personal thoughts and emotions. As such, the relational benefits posited by the IESD framework naturally extend to active usage. Specifically, by facilitating online self-disclosure, ASMU fosters interpersonal intimacy and perceived social support, helping compensate for the loss of informal workplace interactions under remote work and thereby buffering its positive link with loneliness.

In contrast, PSMU consists primarily of observing others’ posts, profiles, and interactions without participating directly in interpersonal exchanges. With few opportunities for self-disclosure, PSMU is unlikely to elicit the reciprocal responses through which trust, intimacy, and perceived social support develop (Frison and Eggermont, 2020; Taylor et al., 2024). It is therefore less likely to activate the relational mechanism specified by the IESD framework and is thus unlikely to buffer the loneliness associated with remote work. Instead, frequent PSMU may even compound loneliness. First, sustained exposure to others’ social activities may heighten users’ awareness of their peripheral position within a social network, producing “crowded loneliness,” a paradoxical condition of being socially or digitally surrounded by others while remaining unable to form emotionally meaningful connections (Karayaman and Deniz, 2026). Second, exposure to others’ selectively curated self-presentations may encourage upward social comparison, generating negative self-evaluations and feelings of relative deprivation. Consistent with these two mechanisms, evidence from both experimental and longitudinal studies links passive social media use to negative relational and emotional outcomes (Roberts and David, 2023; Roberts et al., 2026). Therefore, by intensifying feelings of peripheral exclusion and relative deprivation, high PSMU magnifies the relational vulnerability of remote workers. Taken together, these arguments lead to the following hypotheses:

*H2a*: As the frequency of remote work increases, individuals with higher levels of active social media use will experience a weaker increase in loneliness.

*H2b*: As the frequency of remote work increases, individuals with higher levels of passive social media use will experience a stronger increase in loneliness.

## 3 Method

### 3.1 Data

This study uses secondary data from the United Kingdom Household Longitudinal Study (UKHLS), an ongoing annual household panel survey that began in 2009 and follows individuals living in sampled households across the United Kingdom. Detailed information on the study design, sampling procedures, and fieldwork is provided in the UKHLS Main Survey User Guide and the relevant wave-specific technical reports (Institute for Social and Economic Research, 2026). Secondary analysis of large-scale panel data is a well-established approach in social and health research, offering advantages in sample size, representativeness, and the ability to capture within-person change over time (Halaby, 2004; Vartanian, 2010).

**Figure 1:**
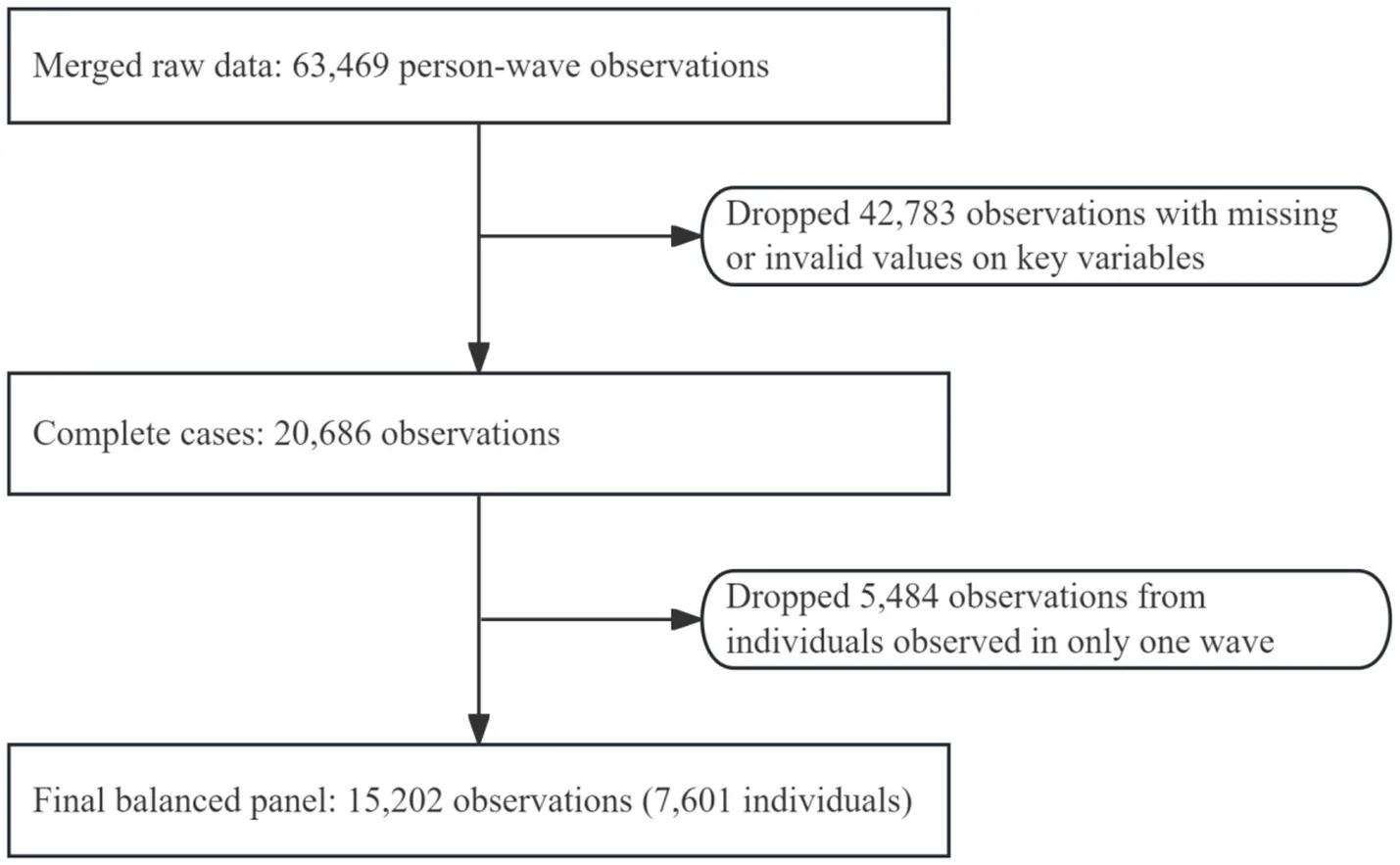
Sample selection process. Of the 42,783 person-wave observations excluded because of missing or invalid values on key variables, 33,994 were excluded due to predictor unavailability. This category included structurally inapplicable cases, such as respondents who were not in paid employment or were self-employed, as well as proxy interviews, refusals, “do not know” responses, and other item nonresponse.

The present analysis draws on Waves 13 (2021–2023) and 14 (2022–2024), the only two UKHLS waves providing a remote-working frequency measure. We focus on employees and exclude non-employees because their employment relationships and workplace contexts are not directly comparable, consistent with previous research (Pauliks, 2025). To ensure all models were estimated on a consistent sample, we excluded observations with missing and invalid values on the dependent variable, independent variable, moderators, control variables, or the alternative dependent variable used in the robustness analyses, and retained only individuals observed in both waves, as fixed-effects estimation relies on within-person change. Figure 1 presents the selection process. The final analytic sample comprises 7,601 individuals (15,202 person-wave observations). Specifically, 44.36% of the observations were from male respondents and 55.64% from female respondents. In terms of age, 15.43% were aged 17–29, 31.34% were aged 30–44, 40.80% were aged 45–59, and 12.43% were aged 60 or above.

### 3.2 Measurement

Remote work frequency was constructed using two items from the UKHLS Employees module: *jbpl* and *jbwah* (Wave 13 questionnaire, pp. 511-512; Wave 14 questionnaire, pp. 595-596). First, employees were asked about their primary work location (“Do you work mainly…?”), choosing from (1) at home, (2) at the employer’s premises, (3) driving or traveling around, or (4) at one or more other places. Those selecting options (2)–(4) were subsequently asked how often they worked at home in their main job, with response categories: “never,” “sometimes,” “often,” or “always.” Based on both items, we constructed a new variable ranging from 1 (never) to 4 (always), where higher values indicate greater remote work frequency. Respondents whose primary location was “at home” were coded as “always.”

Loneliness was assessed using the three-item UCLA Loneliness Scale in the UKHLS Self-Completion Loneliness Module. The three items correspond to variables *sclackcom*, *scleftout*, and *scisolate* (Wave 13 questionnaire, pp. 740–742; Wave 14 questionnaire, pp. 787–788). Respondents were asked how often they feel (1) a lack of companionship, (2) left out, and (3) isolated from others. Responses were rated on a three-point scale: 1 = “hardly ever or never,” 2 = “some of the time,” and 3 = “often.” Scores were summed to create a composite index ranging from 3 to 9, with higher values indicating greater loneliness (Cronbach’s alpha = 0.87).

Two dimensions of social media use were measured using items from the UKHLS Device Use and Online Activity Module, which assessed the frequency of online activities undertaken for personal purposes. Passive social media use was measured using the variable *smlook*, whereas active social media use was measured using *smpost* (Wave 13 questionnaire, pp. 204–205; Wave 14 questionnaire, pp. 244–245). Consistent with the conceptual distinction outlined in Section 2.2, PSMU was assessed by asking how often respondents look at content (e.g., text, images, or videos) on social media platforms such as Facebook, Twitter, or Instagram. ASMU was measured by asking how often respondents post content (e.g., text, images, or videos) on social media platforms such as Facebook, Twitter, or Instagram. Both items used a six-point scale ranging from 1 (“every day”) to 6 (“never”). Both variables were reverse-coded so that higher scores reflect more frequent engagement, facilitating interpretation in the analysis. The measurement of social media “looking” (passive use) and “posting” (active use) behaviors has been widely adopted in prior studies to capture distinct modes of engagement (Yu et al., 2024).

The study controlled for a range of individual-level factors shown to influence loneliness (Barjaková et al., 2023). Consistent with the fixed-effects model specification (see the analytic strategies section), only variables that vary over time were included. These controls comprised age, education level (low, middle, and high), personal income (log), marital status (single, married, and divorced/widowed), household size, physical and mental health status (measured by the 12-Item Short Form Health Survey), employment sector (public and private), and weekly working hours.

### 3.3 Analytical strategies

We first conducted descriptive analyses to summarize respondents’ sociodemographic characteristics and key variables. Subsequently, we used fixed-effects regressions to examine the association between remote work frequency and loneliness. This approach compares the loneliness ratings of the same individual across different levels of remote work engagement. Finally, we tested the differential effects of ASMU and PSMU using interaction terms.

## 4 Results

As shown in Table 1, the mean remote work frequency is 2.10, and the mean loneliness score is 4.32. Passive social media use (mean = 5.36) exceeds active use (mean = 3.17). The sample predominantly consists of middle-aged (mean = 44.72), married (58.31%), and highly educated (53.89%) private sector employees working approximately 33.28 h per week.

**Table 1:** Descriptive statistics of key variables.

Continuous variables	Mean	Standard deviation
Remote work frequency	2.10	1.26
Loneliness	4.32	1.61
Passive social media use frequency (PSMU)	5.36	1.39
Active social media use frequency (ASMU)	3.17	1.73
Age	44.72	12.66
Personal income (log)	7.61	0.53
Physical health status	52.65	8.02
Mental health status	47.32	10.22
Household size	3.01	1.32
Weekly working hours	33.28	9.94

Categorical variables	Frequency	Percent (%)
Marital status		
Single	4,624	30.42
Married	8,864	58.31
Divorced/widowed	1,714	11.27
Employment sector		
Public	6,406	42.14
Private	8,796	57.86
Education level		
Low	5,242	34.48
Middle	1,767	11.62
High	8,193	53.89

Table 2 (Model 1) presents the baseline fixed-effects results. Remote work frequency is positively associated with loneliness (*β* = 0.059, *p* < 0.01), confirming that increased remote work predicts higher loneliness, supporting H1.

**Table 2:** Fixed-effects regression on remote work frequency and loneliness.

Variable	Model 1
	Baseline fixed-effects model
	*β* [95% CI]
Remote work frequency	0.059** [0.018, 0.100]
Age	−0.098 [−0.244, 0.049]
Education level (ref: low)	
Middle	−0.101 [−0.618, 0.416]
High	−0.173 [−0.657, 0.311]
Personal income (log)	0.026 [−0.053, 0.105]
Marital status (ref: single)	
Married	−0.234* [−0.423, −0.045]
Divorced/widowed	−0.200 [−0.457, 0.058]
Household size	0.034 [−0.027, 0.096]
Physical health status	−0.010*** [−0.015, −0.005]
Mental health status	−0.043*** [−0.047, −0.040]
Employment sector (ref: private)	
Public	−0.031 [−0.142, 0.080]
Weekly working hours	−0.002 [−0.007, 0.002]
Constant	11.202*** [4.611, 17.794]
Within *R*-squared	0.078
Number of person-wave observations	15,202

*p < 0.05; **p < 0.01; ***p < 0.001; 95% CI, 95% confidence interval.

**Figure 2:**
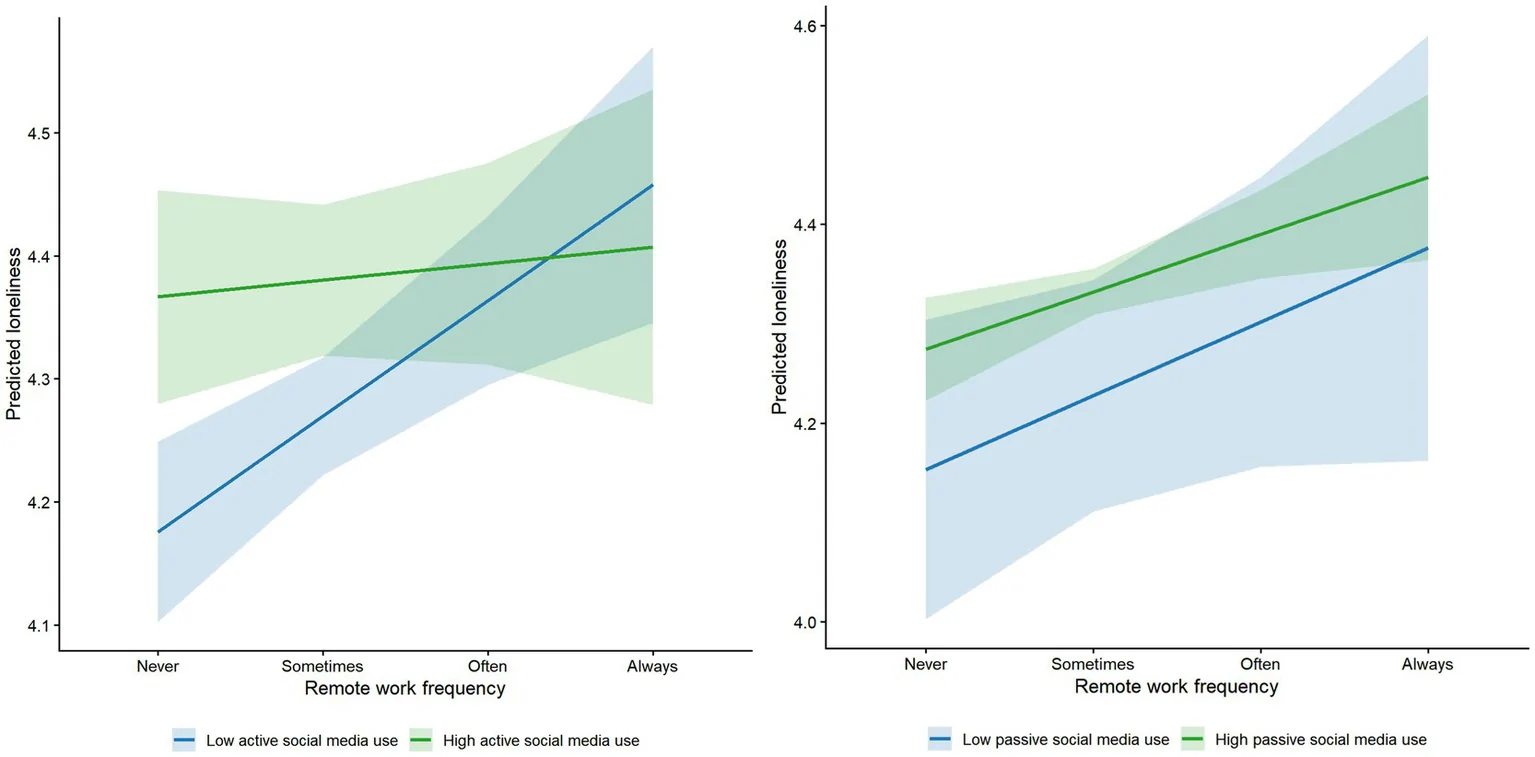
Predicted loneliness by remote work frequency at different levels of active and passive social media use. Low and high social media use correspond to “never” and “every day,” respectively. Shaded areas represent 95% confidence intervals.

To test the proposed moderating effects, we estimated regression models utilizing interaction terms between remote work frequency and each type of social media use. As shown in Table 3 (Model 2), ASMU significantly moderated the relationship between remote work and loneliness (*β* = −0.016, *p* < 0.05), supporting H2a. Figure 2 (left panel) illustrates this pattern, showing a flatter slope for high-ASMU users compared to the steep rise in loneliness for low-ASMU users. In contrast, Model 3 indicates that PSMU did not significantly interact with remote work (*β* = −0.003, *p* > 0.05), thus providing no support for H2b. The broadly similar slopes in Figure 2 (right panel) further demonstrate that PSMU did not significantly moderate the association between remote work frequency and loneliness. Thus, only ASMU showed a statistically significant buffering association.

**Table 3:** Fixed-effects regression examining the interactions between remote work frequency and social media use.

Variable	Model 2	Model 3
	Active social media use (ASMU)	Passive social media use (PSMU)
	*β* [95% CI]	*β* [95% CI]
Remote work frequency	0.110*** [0.047, 0.173]	0.078 [−0.031, 0.186]
Passive social media use frequency	—	0.028 [−0.018, 0.073]
Active social media use frequency	0.054** [0.018, 0.091]	—
Remote work frequency × passive social media use frequency	—	−0.003 [−0.022, 0.015]
Remote work frequency × active social media use frequency	−0.016* [−0.031, −0.001]	—
Confounding variables	Control	Control
Constant	10.820** [4.225, 17.414]	11.075** [4.479, 17.671]
Within *R*-squared	0.079	0.078
Number of person-wave observations	15,202	15,202

*p < 0.05; **p < 0.01; ***p < 0.001; 95% CI, 95% confidence interval.

To ensure the robustness of our findings, we conducted several robustness checks (see Table 4). First, we replaced the UCLA loneliness scale with a single-item self-perceived measure of loneliness (“How often do you feel lonely?”) in the UKHLS to evaluate whether our results depend on the operationalization of the outcome variable. The estimates (Model 4) remained substantively unchanged, indicating that the association between remote work and loneliness is not sensitive to the measurement strategy.

**Table 4:** Robustness checks for the effect of remote work frequency on loneliness.

Variable	Model 4	Model 5	Model 6
	Alternative outcome specification	Alternative key predictor specification	Random-effects specification
	*β* [95% CI]	*β* [95% CI]	*β* [95% CI]
Remote work frequency	0.020* [0.003, 0.036]	—	0.037*** [0.017, 0.058]
Remote work frequency (ref: low)			
High	—	0.142** [0.049, 0.235]	—
Age	0.009 [−0.050, 0.068]	−0.101 [−0.248, 0.045]	0.001 [−0.002, 0.003]
Education level (ref: low)			
Middle	−0.175 [−0.383, 0.033]	−0.096 [−0.613, 0.421]	−0.063 [−0.154, 0.028]
High	−0.031 [−0.226, 0.164]	−0.165 [−0.649, 0.319]	0.051 [−0.011, 0.114]
Personal income (log)	0.014 [−0.018, 0.046]	0.027 [−0.052, 0.106]	−0.037 [−0.088, 0.013]
Marital status (ref: single)			
Married	−0.133*** [−0.209, −0.057]	−0.232* [−0.421, −0.043]	−0.427*** [−0.496, −0.358]
Divorced/Widowed	−0.111* [−0.215, −0.008]	−0.198 [−0.456, 0.060]	0.157** [0.059, 0.254]
Household size	0.004 [−0.021, 0.029]	0.033 [−0.028, 0.095]	−0.020 [−0.041, 0.002]
Physical health status	−0.003*** [−0.005, −0.002]	−0.010*** [−0.015, −0.005]	−0.021*** [−0.024, −0.018]
Mental health status	−0.016*** [−0.018, −0.015]	−0.043*** [−0.047, −0.040]	−0.070*** [−0.072, −0.068]
Employment sector (ref: private)			
Public	−0.017 [−0.062, 0.028]	−0.032 [−0.143, 0.079]	0.017 [−0.034, 0.068]
Weekly working hours	−0.000 [−0.002, 0.002]	−0.002 [−0.007, 0.002]	−0.000 [−0.003, 0.002]
Constant	1.960 [−0.691, 4.612]	11.431*** [4.841, 18.021]	9.210*** [8.812, 9.609]
Within *R*-squared	0.070	0.077	0.075
Number of person-wave observations	15,202	15,202	15,202

*p < 0.05; **p < 0.01; ***p < 0.001; 95% CI, 95% confidence interval.

Second, we redefined the predictor by dichotomizing remote work frequency at the sample mean, dividing respondents into high- versus low-frequency remote workers. This specification (Model 5) tests whether the main findings are driven by the linear treatment of remote work frequency. The results continued to show a positive association between remote work frequency and loneliness, lending further support to the robustness of our main effect.

Third, we estimated random-effects models to incorporate between-individual variation and allow the inclusion of time-invariant factors. As shown in Model 6, the direction and statistical significance of the coefficient for remote work frequency remained consistent with those from the fixed-effects models. Although the Hausman test favored the fixed-effects specification, the alignment of results across both models reinforces the stability of our findings under alternative modeling assumptions.

## 5 Discussion and conclusion

Using nationally representative longitudinal data from the United Kingdom, our study examines how remote work frequency shapes employees’ experiences of loneliness and how different patterns of social media use condition this relationship. We have three major findings.

First, our findings indicate that a higher frequency of remote work is associated with greater loneliness among employees. This result is consistent with Jahoda’s latent deprivation theory and previous empirical evidence (He et al., 2026; Knight et al., 2022). It therefore underscores the need for organizations to address the psychosocial challenges associated with remote work in the post-pandemic workplace. Organizations could provide high-frequency remote workers with targeted training on coping with loneliness and managing stress to strengthen their psychological resilience. They could also establish peer-support arrangements and facilitate regular informal online interactions, thereby providing opportunities for timely emotional support, strengthening employees’ sense of belonging, and mitigating the adverse effects of frequent remote work. However, the relationship between remote work and loneliness should be further examined from a temporal perspective. According to Social Information Processing theory, computer-mediated communication is not inherently incapable of conveying interpersonal warmth; rather, compared with face-to-face interaction, it may just require more time for relational cues to accumulate and interpersonal closeness to develop (Walther, 1995). Future research could therefore consider the duration of employees’ remote work experience as a potential boundary condition.

Second, by introducing ASMU as a moderator, this study provides a nuanced understanding of when remote work frequency exacerbates employees’ loneliness, advancing the current literature. Recent research has identified dog caregiving and outdoor access as moderators of the relationship between remote work and loneliness (Delanoeije and Verbruggen, 2024), whereas our study extends this line of inquiry to online social interaction. Our finding is consistent with the IESD hypothesis. ASMU involves greater interpersonal engagement and more frequent opportunities to disclose personal thoughts, feelings, and experiences. Such self-disclosure may elicit emotional support and responsive feedback from others, enhance relational closeness, and reduce the discrepancy between employees’ desired and actual social relationships. In turn, these relational benefits may help attenuate the loneliness associated with frequent remote work. The significant moderating effect identified in our study is also consistent with previous evidence showing that active and interaction-oriented social media use can reduce loneliness, including research on older adults’ active social media use (Yang et al., 2021) and an experimental study demonstrating that increased Facebook status updating reduced loneliness (Deters and Mehl, 2013). Relatedly, drawing on the far-but-close paradox, Labrado-Antolín et al. (2025) found that among remote workers, effective enterprise social network use, together with communication and coworker identification, strengthened perceived proximity and, in turn, affective well-being. This parallels our findings by suggesting that mediated communication protects well-being primarily when it is used actively to sustain relational closeness. Based on these findings, encouraging remote workers to use social media more actively, such as sharing life moments, commenting on others’ posts, and participating in online communities, may help alleviate feelings of loneliness. However, this recommendation should not be interpreted as encouraging remote workers to spend more time on social media indiscriminately during working hours. Prolonged and habitual social media use may interrupt concentration, blur the boundary between work and non-work activities, and contribute to cyberloafing, with potential costs for individual and organizational productivity (Chakraborty et al., 2024). Organizations should therefore encourage bounded and purposeful forms of ASMU during designated breaks or periods of lower task demand (Albulescu et al., 2022).

Third, contrary to our expectations, PSMU did not significantly moderate the association between remote work frequency and loneliness. Although this hypothesis was not supported, the null finding is broadly consistent with previous evidence suggesting that relationships involving PSMU are not always statistically significant. For instance, Yang et al. (2021) reported no significant association between PSMU and loneliness, while Mao and Zhang (2023) also found that PSMU did not significantly moderate the association between ASMU and state fear of missing out. One possible explanation is that the socially distancing nature of remote work is sufficiently strong that any additional influence of passive browsing is overshadowed. In this context, PSMU may function primarily as a low-effort form of distraction or entertainment, without substantially altering the baseline association between reduced offline interactions and loneliness. Another possible explanation lies in differences in how PSMU is experienced in different contexts. Recent meta-analytic evidence indicates that the negative effects of PSMU on well-being vary across contexts, such as whether passive use occurs within social media groups (Godard and Holtzman, 2024). Individuals who work remotely may be more likely to join online group chats to obtain information. These groups often consist of members who share similar experiences and concerns, which may reduce upward social comparison and feelings of exclusion. In such contexts, even passive consumption may not further intensify loneliness, potentially explaining the non-significant moderating effect observed. A third possibility concerns the restorative function of passive use. Brief, hedonic social media browsing may operate as a low-effort micro-break that temporarily redirects attention away from demanding work tasks (Grobelny et al., 2024). When these modest restorative benefits coexist with the envy and social-comparison costs associated with passive browsing, the opposing mechanisms may offset one another, resulting in a null net moderating effect of PSMU.

Taken together, our findings can be understood in light of two apparently competing perspectives on digitally mediated connection: the far-but-close paradox and the crowded loneliness paradox. The far-but-close paradox suggests that physical separation need not produce psychological distance when mediated communication promotes reciprocal interaction, mutual understanding, and a shared sense of identification (Wilson et al., 2008). By contrast, the crowded loneliness paradox cautions that individuals may remain emotionally disconnected despite being surrounded by numerous online contacts when those connections are superficial, unresponsive, or relationally unfulfilling (Karayaman and Deniz, 2026). These perspectives together suggest that the relational consequences of digital communication depend on how, rather than merely how much, individuals interact online. The buffering effect of ASMU is consistent with the far-but-close paradox because active use involves self-disclosure, interpersonal exchange, and opportunities for responsive feedback, which may help transform physical distance into relational closeness. PSMU, however, provides social exposure without necessarily generating reciprocal engagement or perceived proximity, which may explain why it did not buffer the association between remote work frequency and loneliness.

Despite these theoretical and empirical contributions, this study has several limitations that should be acknowledged. First, although we used longitudinal data, only two waves were included due to concerns about measurement consistency across waves. This limits our ability to capture longer-term within-individual change. Future studies should trace temporal dynamics better. Second, active and passive social media use were measured using single-item frequency indicators, which may limit construct precision. Recently, several validated scales have been developed to assess different dimensions of ASMU and PSMU (Niu et al., 2025). Future studies could adopt these scales to improve measurement accuracy and reliability. Third, while the fixed-effects model reduces concerns about time-invariant unobserved heterogeneity, it cannot entirely rule out omitted variables or reverse causality. Thus, our findings should not be interpreted as strictly causal. Future work should apply stronger identification strategies, such as instrumental variables or experimental designs. Finally, our analysis is confined to the United Kingdom context, and the generalizability of the findings to other cultural and institutional settings remains uncertain. Countries differing in remote work adoption, technological infrastructure, or organizational support for flexible working arrangements may exhibit distinct patterns. Future research could examine whether these associations replicate across diverse national contexts.

## Data Availability

Publicly available datasets were analyzed in this study. This data can be found at: https://www.understandingsociety.ac.uk/.
